# Social structure of perennial *Vespula squamosa* wasp colonies

**DOI:** 10.1002/ece3.8569

**Published:** 2022-02-10

**Authors:** Carl J. Dyson, Henry G. Crossley, Charles H. Ray, Michael A. D. Goodisman

**Affiliations:** ^1^ School of Biological Sciences Georgia Institute of Technology Atlanta Georgia USA; ^2^ 1383 Department of Entomology and Plant Pathology Auburn University Auburn Alabama USA

**Keywords:** cytochrome b, DNA microsatellite, eusocial, hymenoptera, polyandry, polygyne, reproductive conflict, yellowjacket wasp

## Abstract

Many social species show variation in their social structure in response to different environmental conditions. For example, colonies of the yellowjacket wasp *Vespula squamosa* are typically headed by a single reproductive queen and survive for only a single season. However, in warmer climates, *V. squamosa* colonies sometimes persist for multiple years and can grow to extremely large size. We used genetic markers to understand patterns of reproduction and recruitment within these perennial colonies. We genotyped *V. squamosa* workers, pre‐reproductive queens, and males from perennial colonies in the southeastern United States at 10 polymorphic microsatellite loci and one mitochondrial DNA locus. We found that *V. squamosa* from perennial nests were produced by multiple reproductives, in contrast to typical annual colonies. Relatedness of nestmates from perennial colonies was significantly lower than relatedness of nestmates from annual colonies. Our analyses of mitochondrial DNA indicated that most *V. squamosa* perennial colonies represented semiclosed systems whereby all individuals belonged to a single matriline despite the presence of multiple reproductive females. However, new queens recruited into perennial colonies apparently mated with non‐nestmate males. Notably, perennial and annual colonies did not show significant genetic differences, supporting the hypothesis that perennial colony formation represents an instance of social plasticity. Overall, our results indicate that perennial *V. squamosa* colonies show substantial changes to their social biology compared to typical annual colonies and demonstrate variation in social behaviors in highly social species.

## Graphical abstract

Social species show variation in their social behaviors in response to environmental differences. We found that the social wasp *Vespula squamosa* displayed remarkable variation in social behavior that resulted in extreme changes to colony structure and ontogeny. This shift in social strategy may help wasp societies survive and dominate the local environment when subject to variable conditions.

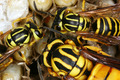

## 1 INTRODUCTION

The evolution of advanced societies represented an important and successful major transition in biological history (Maynard Smith & Szathmary, 1998; Wilson, 1971). The most remarkable of these societies are displayed by social insects, which include ants, termites, social bees, and social wasps. Social insects play major roles in many ecological communities and are considered among the most successful of animal taxa (Wilson, 1987). The success of social insects arises from their use of cooperative and helping behaviors to complete important tasks such as rearing young, defending the colony, and foraging for food. These behaviors, which are critical to the growth and survival of social insect populations, could be affected by changes to outside factors such as environmental conditions. Social insects may respond to such disturbances through behavioral plasticity leading to changes in cooperative actions (Andrew et al., 2013; van Baaren & Candolin, 2018; Czaczkes & Heinze, 2015; Schurch et al., 2016). Such changes could ultimately lead to evolution of the social structures that define insect societies and are of primary importance to their persistence, survival, and success (Hölldobler & Wilson, 1990; Queller & Strassmann, 1998; Ratnieks et al., 2006; Ross & Matthews, 1991).

**FIGURE 1:**
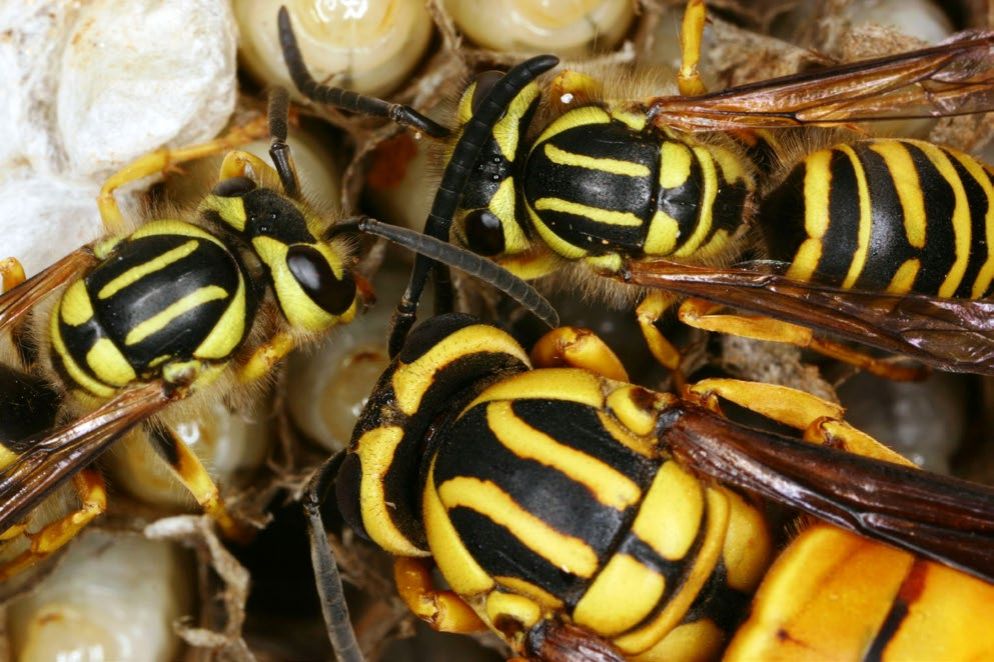
Queen and worker *Vespula squamosa* social wasps inside the nest. This study investigates the genetic structure of *V. squamosa* perennial colonies, which can grow to extreme size and have profound ecological impacts

**FIGURE 2:**
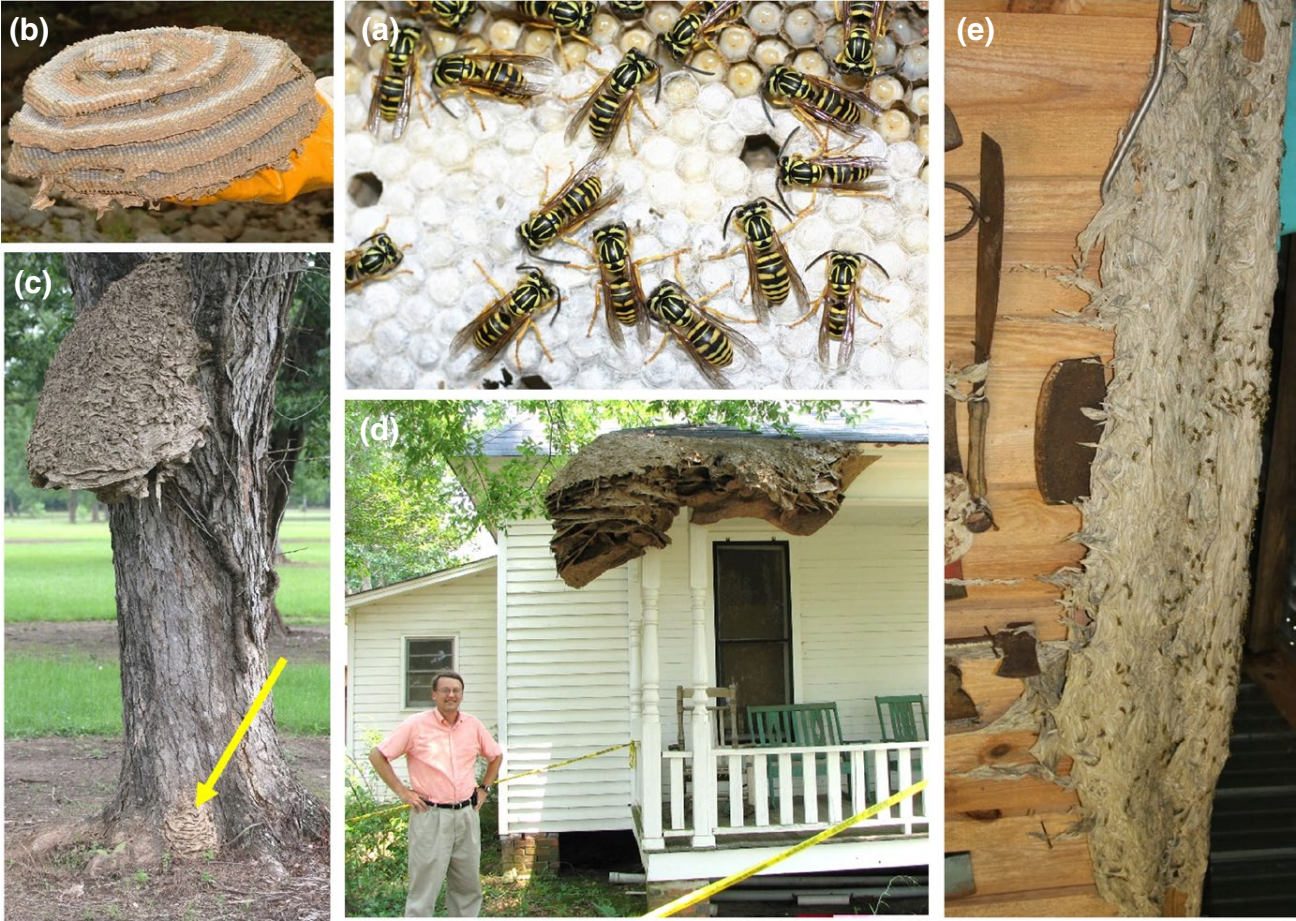
The southern yellowjacket, *Vespula squamosa*. (a) *Vespula squamosa* workers inside of an active colony. (b) Comb from a mature, annual colony of *V. squamosa*. (c) Perennial colony "MO," with small satellite colony at base of tree indicated by arrow. (d) Perennial *V. squamosa* colony in a home. (e) Large perennial *V. squamosa* colony invading structure. Photocredits: Michael Goodisman (a,b) and Charles Ray (c,d,e)

The goal of this study was to understand the potential differences between social systems and behaviors in annual and perennial colonies of a social insect. Specifically, we studied changes in the societies of the highly social wasp, *Vespula squamosa* (Figures 1 and 2a). *Vespula squamosa*, locally known as the southern yellowjacket, is a common social wasp found throughout the Southeast of the United States and extending through Mexico and into Central America (Akre et al., 1980; Hunt et al., 2001; Landolt et al., 2009). Typically, single queens initiate new nests after a period of overwintering (natural history reviewed by Edwards, 1980; Greene, 1991; Spradbery, 1973). The new queen may construct her own incipient nest. Alternatively, newly emerged queens sometimes invade the nests of congeners and take over the already established colony (Allen et al., 2020; MacDonald & Matthews, 1975, 1984). Regardless of the method of nest initiation, the single queen remains wholly responsible for the production of offspring within the nest as long as she is present.

Within‐colony genetic diversity in *V. squamosa* is directly related to queen mate number, as queens of all *Vespula* taxa mate with multiple males (polyandry) and all annual *Vespula* colonies are headed by a single queen (monogyny) (Foster & Ratnieks, 2001; Goodisman, Matthews, & Crozier, 2007; Goodisman, Matthews, Spradbery, et al., 2001; Goodisman et al., 2002, 2007a, 2007b; Hoffman et al., 2008; Kovacs & Goodisman, 2012; Ross, 1986; Wenseleers et al., 2005). A mature *V. squamosa* nest consists of several layers of comb and may contain ~5000 cells used to produce thousands of individual wasps (MacDonald & Matthews, 1984) (Figure 2b). *Vespula squamosa* colonies then produce new queens and males that proceed on mating flights to propagate with reproductives from other colonies. Newly mated queens find locations to hibernate during the winter and the old colony, including all remaining workers, males, and queens, die off as winter approaches.

However, *V. squamosa* colonies sometimes can persist for more than one year (Deets & Fritz, 2002; Ross & Matthews, 1982; Ross & Visscher, 1983). When conditions are favorable, including sufficiently high temperatures and adequate food availability, nests can continue to grow rather than dying off in the winter. These nests can take on extremely large size very quickly, growing exponentially so that after only two years they may be ten times larger than a typical annual nest (Figure 2c‐e). One perennial *V. squamosa* nest contained ~475,000 cells (Pickett et al., 2001), which is 100× larger than a typical, annual nest. The residents of this massive colony were estimated to have consumed ~215 kg of arthropod prey or perhaps 5,000,000 prey items.

This study used genetic approaches to interrogate the social structure of perennial *V. squamosa* colonies. We determined the relationships of nestmates and the reproductive patterns of individuals within colonies. We also investigated the origin of individuals within colonies and possible genetic differences between the annual and perennial social forms. We interpret our results in light of our understanding of the factors that contribute to variation in sociality. Overall, our research provides insight into the evolution and plasticity of social systems in highly social species.

## 2 MATERIALS AND METHODS

### 2.1 Sample collection

Perennial colonies of *V. squamosa* were collected in Alabama, USA, between July 19, 2019, and July 3, 2020. Samples were collected from eight perennial colonies (Table 1). One colony had a small apparent satellite colony that was also sampled for analysis (Figure 2c). For collection, colonies were treated with carbon dioxide or pyrethrin and piperonyl butoxide depending on the location of the nest.

**TABLE 1:** Locations and number of workers, gynes, and males genotyped at nuclear microsatellite (and mitochondrial) markers from perennial and annual *Vespula squamosa* colonies

Social form	Colony ID	Latitude	Longitude	Workers	Gynes	Males	Total
Perennial	HA	33.1175	−86.1227	80 (13)			80 (13)
	KL	32.1253	−85.9370	80 (14)	48 (10)		128 (24)
	MA	33.9681	−87.8050	82 (13)	2 (2)	24 (9)	108 (24)
	MC	33.2197	−86.3014	80 (14)	4 (4)	30 (12)	114 (30)
	MO	32.3855	−86.3099	75 (21)	33 (19)	18 (16)	126 (56)
	SP	32.4669	−87.2127	66 (14)			66 (14)
	ST	31.1180	−87.4632	80 (14)	5 (4)		85 (18)
	WA	31.0482	−87.7152	80 (14)	3 (2)		83 (16)
Total				623 (117)	95 (41)	72 (37)	790 (195)
Annual	2	33.8952	−84.6341	40 (2)			40 (2)
	4	33.9926	−84.2873	11 (2)			11 (2)
	5	33.7916	−84.3741	40 (2)			40 (2)
	6	33.7916	−84.3741	40 (2)			40 (2)
	11	34.5877	−84.0029	40 (2)			40 (2)
	12	34.5895	−84.0051	40 (2)			40 (2)
	21	33.7330	−84.3737	40 (2)			40 (2)
	22	33.7330	−84.3737	40 (2)			40 (2)
	26	34.0933	−84.1946	40 (2)			40 (2)
	31	33.9650	−84.5408	40 (2)			40 (2)
	32	33.9302	−83.3941	40 (1)			40 (2)
	42	34.0027	−84.3816	35 (2)			35 (2)
	48	33.7330	−84.3737	39 (2)			39 (2)
Total				485 (25)			485 (25)
Grand Total				1108 (142)	95 (41)	72 (37)	1275 (220)

Individuals were manually extracted from the nest material. Workers were collected from all colonies. In addition, a small number of presumptive gynes (pre‐reproductive queens) and males were sampled from a subset of colonies. Individuals were preserved in 95% ethanol and transported to the laboratory for genetic analysis.

### 2.2 Laboratory analysis

DNA was extracted from the rear leg of individual wasps using the Chelex^®^ protocol (Walsh et al., 1991). The genotypes of all individuals were determined at ten polymorphic microsatellite loci including the following: LIST‐2003, LIST‐2004, LIST‐2007, LIST‐2008, LIST‐2013, LIST‐2015, LIST‐2019, LIST‐2020, Rufa05, and VMA‐6 (Daly et al., 2002; Foster et al., 2001; Hasegawa & Takahashi, 2002; Hoffman et al., 2008). Additionally, we sequenced a portion of the mitochondrial DNA (mtDNA) of a subset of individuals from each nest using the primers CB1 and CB2 (Chiotis et al., 2000). The mtDNA primers amplified a 458‐bp segment of the cytochrome b gene.

PCRs were used to amplify DNA at the microsatellite loci and the cytochrome b mtDNA locus. PCRs were generally conducted in a final volume of 15 μl composed of: 6.4 μl deionized water, 2.4 μl 25 mM MgCl2, 1.5 μl 10× PCR buffer, 1.2 μl 2.5 mM dNTPs, 1 μl Taq polymerase, 0.75 μl each of 10 μM reverse and fluorescence‐tagged forward primers, and 1 μl of DNA. The PCR amplification profile used for each locus was: 2 min at 94°C, 35 cycles for 30 s at 94°C, 30 s at locus‐specific annealing temperature (Table 2), 30 s at 72°C, and then a final extension for 5.5 min at 72°C. PCR products were run on a 3% agarose gel at 100V to verify amplification for each individual.

**TABLE 2:** Variability and amplification metrics for microsatellite and mtDNA loci used in analysis of perennial *Vespula squamosa* individuals

Locus	A_n_	A_e_	H_e_	H_o_	T_A_ (°C)	Size (bp)
LIST2003	35	9.41	0.894	0.806	55	172–226
LIST2004	15	4.73	0.789	0.812	55	119–160
LIST2007	30	14.1	0.929	0.887	55	141–194
LIST2008	29	7.09	0.859	0.750	55	125–161
LIST2013	26	7.94	0.874	0.879	55	169–205
LIST2015	9	2.79	0.642	0.637	55	162–174
LIST2019	3	1.55	0.356	0.333	60	122–133
LIST2020	21	11.0	0.909	0.861	55	324–415
RUFA5	13	5.67	0.824	0.766	58	154–175
VMA6	29	9.16	0.891	0.867	58	269–303
mtDNA Cytochrome b^a^	5^a^	–	0.564^a^	–	45	458

Abbreviations: A_e_, Effective number of alleles; A_n_, Total number of alleles; H_e_, Expected heterozygosity; H_o_, Observed heterozygosity; Size, Range of amplicon allele sizes; T_A_, PCR primer annealing temperature.

^a^ Number of haplotypes and haplotype diversity for mtDNA marker.

After confirmation of amplification, microsatellite genotypes were analyzed using the fragment analysis module of an ABI 3100 sequencer. Scoring was completed using a combination of GeneMapper v4.0 (Applied Biosystems, Foster City, CA) and manual scoring of peaks. mtDNA amplicons were sequenced using the CB1 primer by Eton Biosciences.

### 2.3 Microsatellite genetic analysis

In total, 790 individuals from eight perennial colonies were newly genotyped in this study (Table 1). In addition, we incorporated data from a prior study of annual *V. squamosa* colonies into this investigation for comparative purposes (Hoffman et al., 2008). This previous study of annual colonies included data from 485 workers from 13 annual colonies, which were collected in and around Atlanta, GA, USA, between July 2004 and July 2005 and were previously genotyped at seven of the ten loci used in this study (Table 1).

Population genetic statistics for each microsatellite locus, including effective and observed number of alleles, as well as expected and observed heterozygosity, were estimated using SPAGEDi (Hardy & Vekemans, 2002). Perennial and annual locus statistics were compared using paired t‐tests and Wilcoxon rank sum tests implemented in JMP Pro 15 (SAS Institute Inc, 2019).

We investigated potential genetic differences between perennial colony “MO” and its putative, small, satellite colony (Figure 2c). We used the program GENEPOP (Rousset, 2008) to determine if the distribution of worker, male, and gyne genotypes differed between the satellite and the parent nest.

Similarly, we used GENEPOP to conduct genotypic probability tests to determine whether the distribution of genotypes of workers sampled from perennial colonies differed from the distribution of gynes sampled from the same colony. Such a result would be expected if the reproductives (queens or males) within colonies contributed differentially to the production of gynes and workers. The resulting *p*‐values from these analyses were then combined across perennial colonies using Stouffer's Z‐transform test to determine the overall significance of genetic differences between castes for all colonies.

We then investigated the distribution of genotypes of workers, gynes, and males within perennial colonies to determine whether individuals of each caste were produced by more than one reproductive queen. For example, the presence of three homozygous genotypes among the workers or gynes, or three different alleles among the males, would indicate that these individuals arose from multiple reproductives. We then used the program COLONY (Jones & Wang, 2010) to provide estimations of parentage and number of queens within each perennial colony.

We estimated the relatedness of nestmate workers, gynes, and males from measures of genetic variability, as determined by the relationship r = 2*F*_ST_ / (1 + *F*_IT_) (Pamilo, 1989). Standard errors of the mean (SEM) were calculated from the locus‐specific relatedness estimates. Significant differences between estimates of relatedness were determined based on whether 95% confidence intervals (1.96 ± SEM) overlapped.

Next, we investigated the putative genetic differences between perennial and annual colonies. First, we compared the number of alleles segregating within perennial colonies to the number of alleles segregating within annual colonies. This provided information on the number and origin of reproductives within colonies of the different social forms.

Then, we investigated whether the perennial and annual *V. squamosa* colonies displayed genetic isolation by distance. We first calculated paired *F*_ST_ values between all colonies using GENEPOP. Pairwise geographic distances were calculated between each of the colonies using GenAlEx 6.5 (Peakall & Smouse, 2012). We then used a Mantel test within GENEPOP and Spearman's rank correlation coefficient (*r_s_*) to determine the significance of the correlation between geographic and genetic distance.

Finally, we examined genetic differences between perennial and annual colonies of *V. squamosa* using the program GDA (Lewis & Zaykin, 2000). *Vespula* species within their native ranges do not show significant variation in allele frequency and genetic diversity over time (Dyson et al., 2021). Thus, analysis of genetic differences between *V. squamosa* annual and perennial colonies in this study would provide a rudimentary test of genetic differentiation between social forms. We used a hierarchical analysis of genetic structure to measure genetic differences between both “social form” (annual or perennial) and “colony within social form.” Estimates of theta for these levels (θ_S_ and θ_C_, respectively) provided information of genetic differentiation of social forms, while controlling for genetic differences between colonies.

### 2.4 Mitochondrial genetic analysis

We obtained the sequence of 223 individuals from 8 perennial and 13 annual colonies at the PCR‐amplified cytochrome b fragment (Table 1). Sequences from both the perennial and annual colonies were newly obtained in this study. Quality assessment of unassembled mtDNA sequences was performed manually. The program CHROMAS was used to visualize chromatogram output and identify low‐quality or ambiguous base calls for trimming. Sequences for cytochrome b were aligned by MUSCLE (Edgar, 2004). MEGA X was used to trim low‐quality bases from the reads, resulting in a final cytochrome b fragment of 338 bp (Kumar et al., 2018). Polymorphic bases among mtDNA sequences and haplotype diversity were estimated using DnaSP v6 (Rozas et al., 2017).

We generated a phylogeny of the mtDNA haplotypes. In order to create a rooted phylogeny of *V. squamosa* haplotype sequences, additional *Vespula* cytochrome b mtDNA sequences were downloaded from GenBank for inclusion in the analysis. These samples consisted of two additional *Vespula squamosa* sequences (Landolt et al., 2010; Lopez‐Osorio et al., 2014), as well as sequences from two outgroup species, *V. maculifrons* (Landolt et al., 2010; Lopez‐Osorio et al., 2014) and *V. germanica* (Eloff et al., 2020). Phylogenetic relationships were determined using MEGA X and maximum likelihood trees were constructed following the HKY nucleotide substitution model. Trees were tested using the bootstrap method with 500 replications.

Finally, we investigated whether there were differences in mtDNA frequencies between the perennial and annual colonies. We used GENEPOP to determine the significance of differences in haplotype frequency between social forms. Due to the lack of intracolony haplotype diversity within our samples, we collapsed our dataset so that information from each colony was represented by only a single individual. We then analyzed this reduced dataset consisting of 21 individuals (8 perennial and 13 annual) for differences between social forms using a probability test.

## 3 RESULTS

### 3.1 Microsatellite genetic analysis

We identified one perennial colony, MO, that apparently had developed a small satellite colony near it (Figure 2c). A probability test of genotypic differentiation was used to determine whether individuals sampled from the parent nest and satellite nest differed genetically. The genotypes of workers, gynes, and males sampled from the parent and satellite nest showed substantial but nonsignificant differences (*p* = .051, *p* = .9685, and *p* = .0584, respectively). Thus, we combined individuals from the MO parent and satellite nest together for all subsequent analyses.

We next investigated whether the distribution of gyne and worker genotypes within perennial colonies differed significantly. Our analysis uncovered evidence that the distribution of gyne genotypes differed significantly from that of workers in 3 of the 6 colonies (Table 3). We then used Stouffer's Z‐transform analysis to provide a secondary test of significance across all six colonies and found that the differences in genotypic distributions were highly supported (*p* < .001). Thus, there is some evidence of genetic differences between castes in perennial colonies.

**TABLE 3:** Tests of genotypic differentiation between gynes and workers in six perennial *Vespula squamosa* colonies and for all colonies combined

Colony	*N*_G_	*N*_W_	χ^2^	*df*	*p*
KL	48	80	31.64	20	.0473*
MA	2	82	40.19	18	.0020**
MC	4	80	16.23	20	.7022
MO	75	33	77.25	16	.0001***
ST	5	80	18.34	20	.5648
WA	3	80	20.66	20	.4172
All					.0001***

Note

All, Combined *p*‐value from Z‐transform analysis; *df*, Degrees of freedom; *N*_G_, Number of gynes; *N*_W,_ Number of workers; *p*‐value, *p* with *p < .05, **p < .01, ***p < .0001; *χ* ^2^, Chi‐square statistic.

The genotypes of workers, gynes, and males within perennial colonies were analyzed to determine whether they were consistent with having been produced by more than a single queen. A single‐queen model would demand that the genotype of every individual include one of the colony queen's two alleles. We found that individuals from perennial colonies could not have been produced by a single queen in all colonies and for all castes. Moreover, iterative maximum‐likelihood reconstructions of family relationships by the program COLONY produced a mean estimation of queen number of 21 for perennial colonies (colony HA: 14, KL: 29, MA: 9, MC: 21, MO: 25, SP: 18, ST: 32, WA: 20).

**FIGURE 3:**
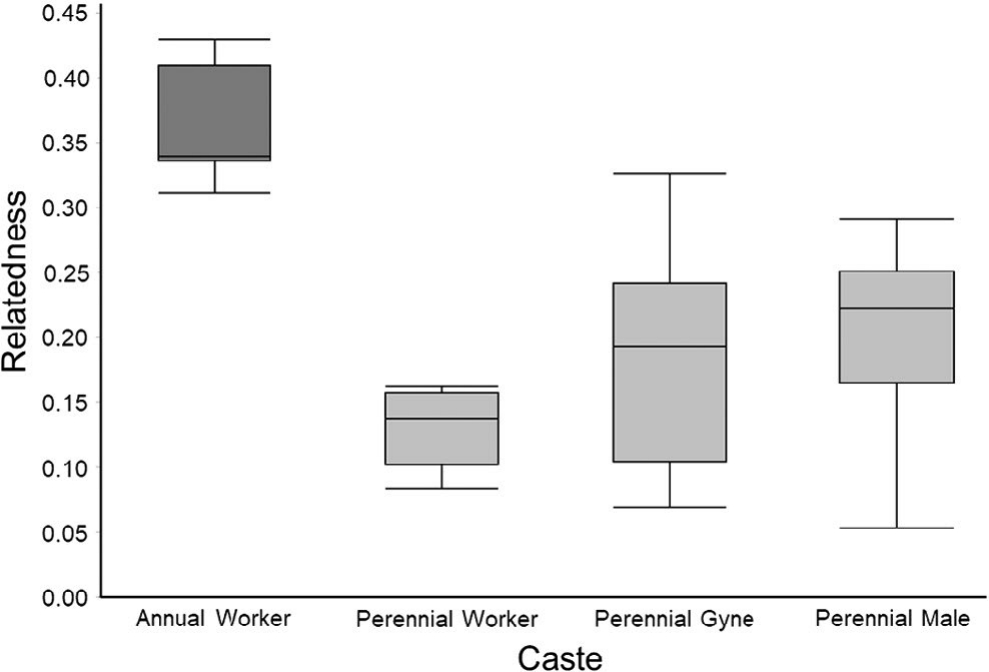
Relatedness between individuals of different castes in perennial and annual *Vespula squamosa* colonies. Boxes display first quartile, median, and third quartile values, whereas whiskers represent values within 1.5X the interquartile range. Workers sampled from annual colonies were significantly more highly related than those sampled from perennial colonies

We next calculated the relatedness of nestmate workers, gynes, and males from perennial colonies (Figure 3). We found that the relatedness of perennial workers (0.130 ± 0.0095) was significantly lower than that of annual workers (0.368 ± 0.0164). The relatedness of gynes in perennial nests was 0.179 ± 0.0262, which did not differ significantly from the estimate for perennial workers. The relatedness of males from perennial colonies was 0.211 ± 0.0216, which was significantly below the value of 0.5 expected if males were produced by a single queen.

**FIGURE 4:**
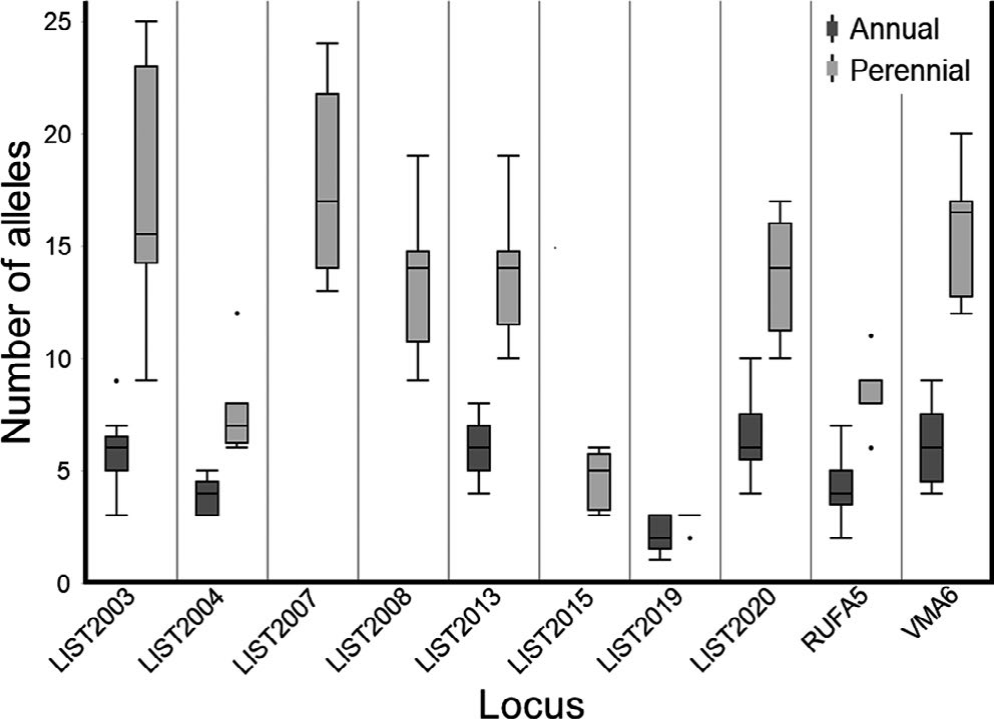
Number of alleles segregating at microsatellite loci in perennial and annual social forms of *Vespula squamosa*. Boxes display first quartile, median, and third quartile values, whereas whiskers represent values within 1.5X the interquartile range. Overall, perennial colonies showed a significantly higher number of alleles than annual colonies

We next investigated genetic differences between the perennial and annual *V. squamosa* social forms. Our analyses revealed that the mean number of alleles present within perennial *V. squamosa* individuals was double to triple the mean number of alleles in annual colonies at 6 of the 7 loci and differed significantly between social forms overall (paired *t*‐test, *t* = −2.555, *p* = .0432; Figure 4). Specifically, the number of alleles in perennial colonies was significantly greater than those in annual colonies for all loci (Wilcoxon 2‐sample test, *p* < .001) except for LIST2019 (*p* = .0541). The effective number of alleles, however, did not differ significantly between the social forms, suggesting that many of the observed alleles in perennial colonies were present at relatively low frequency (paired *t*‐test, *t* = −2.284, *p* = .0625).

**FIGURE 5:**
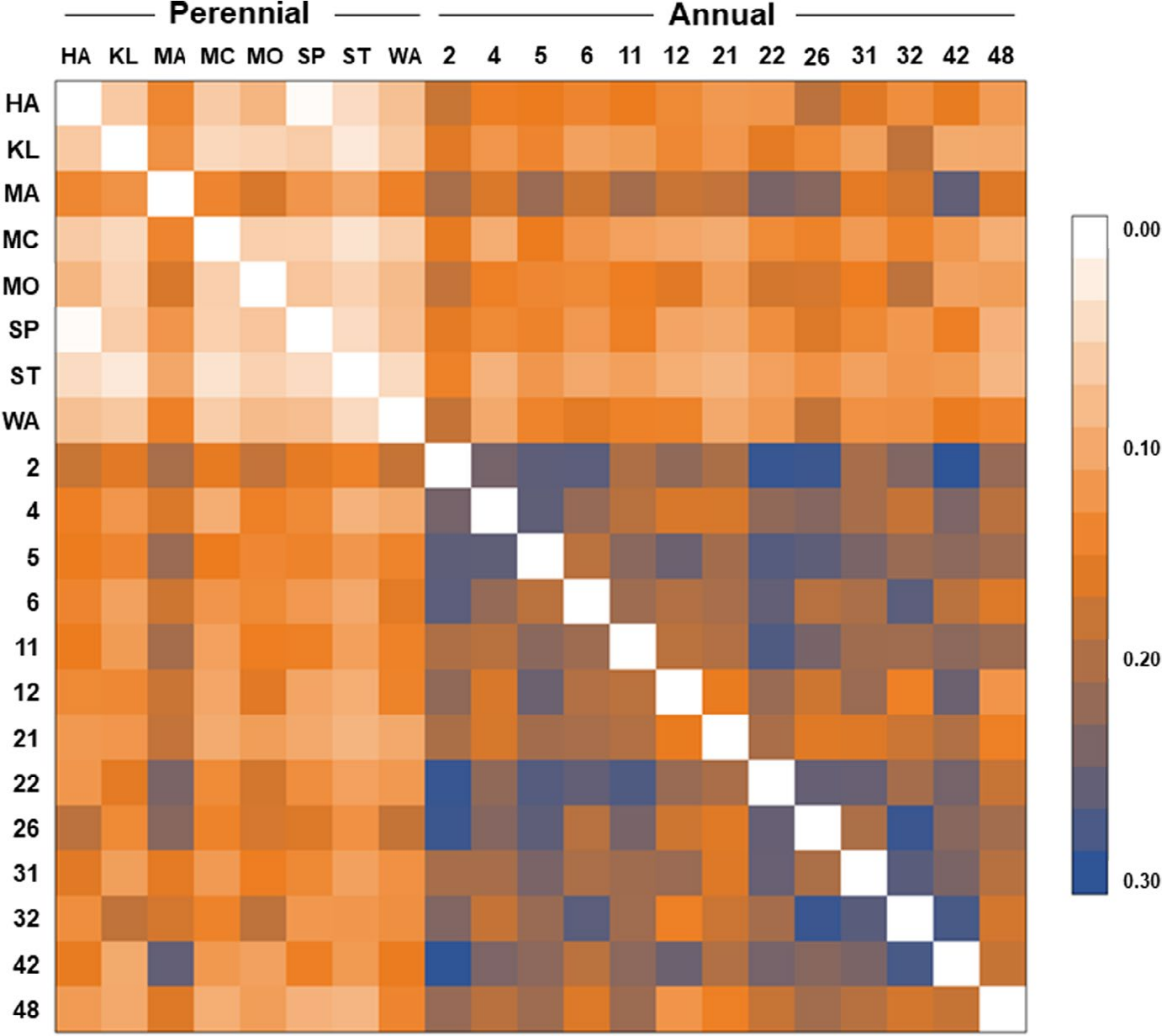
Pairwise *F*_ST_ values between all *Vespula squamosa* colonies. Annual colonies (denoted by numerical labels; *N* = 13) displayed a higher degree of differentiation between colonies than perennial colonies (denoted by two‐letter name of collection site; *N* = 8)

Genetic differences between colonies were estimated by calculating *F*_ST_ between all pairs of colonies (Figure 5). The *F*_ST_ values for the annual colonies were generally substantially higher than those for the perennial colonies (*t*‐test, *t* = −11.1438, *p* < .001). Moreover, the pairwise *F*_ST_ values for intersocial form comparisons were more moderate and fell between the values for the annual and perennial colonies.

We used Mantel tests to investigate genetic isolation by distance for the annual and perennial colonies. Annual colonies, perennial colonies, and all colonies combined showed no evidence of genetic isolation by distance across their respective collection ranges (*p* = .404, *p* = .541, *p* = .995, respectively). That is, colonies that were more distantly separated geographically from each other did not display significantly more genetic differentiation due to distance. Thus, overall, we find no evidence of population genetic isolation in *V. squamosa*.

**FIGURE 6:**
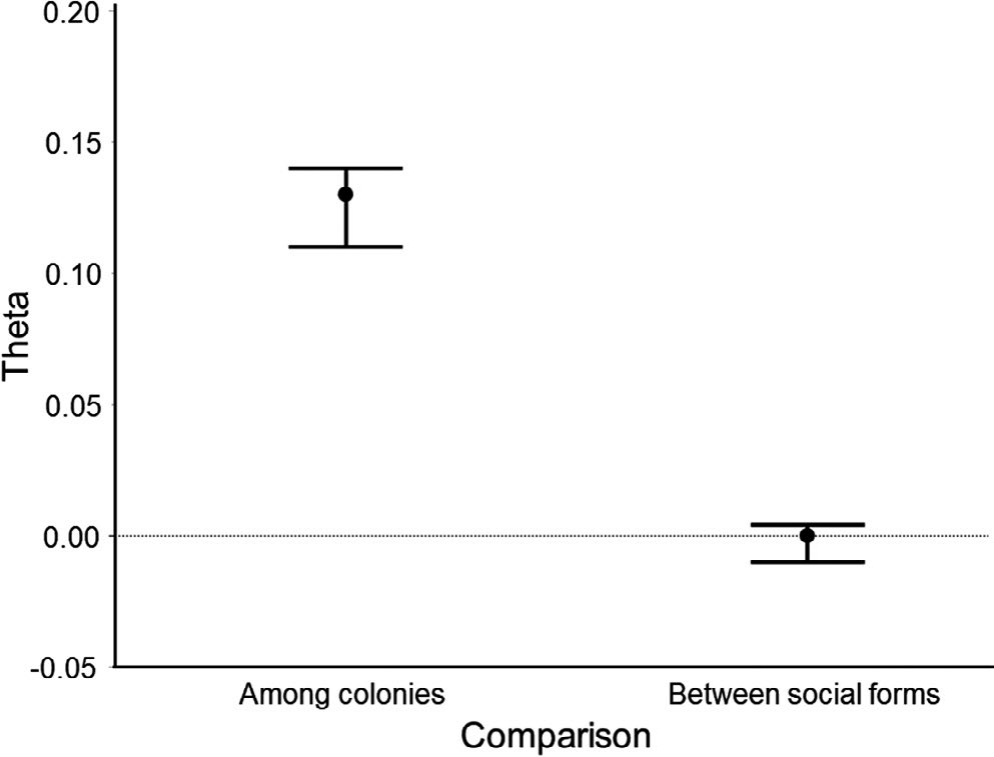
Means and 95% confidence intervals for comparisons of genetic differentiation among colonies within social forms (θ_c_) and between social forms (θ_S_)

Genetic differences between the perennial and annual social forms were further investigated using a hierarchical analysis of genetic structure. In particular, we were interested in understanding genetic differences between social forms while controlling for differences between colonies within social forms. The hierarchical analysis of variance of the nuclear microsatellite markers revealed no evidence of genetic differences between social forms (θ_S_). In contrast, there were substantial genetic differences among colonies within social form (Figure 6). Thus, overall, there was no evidence of genetic differentiation between annual and perennial social forms of *V. squamosa*.

### 3.2 Mitochondrial genetic analysis

**FIGURE 7:**
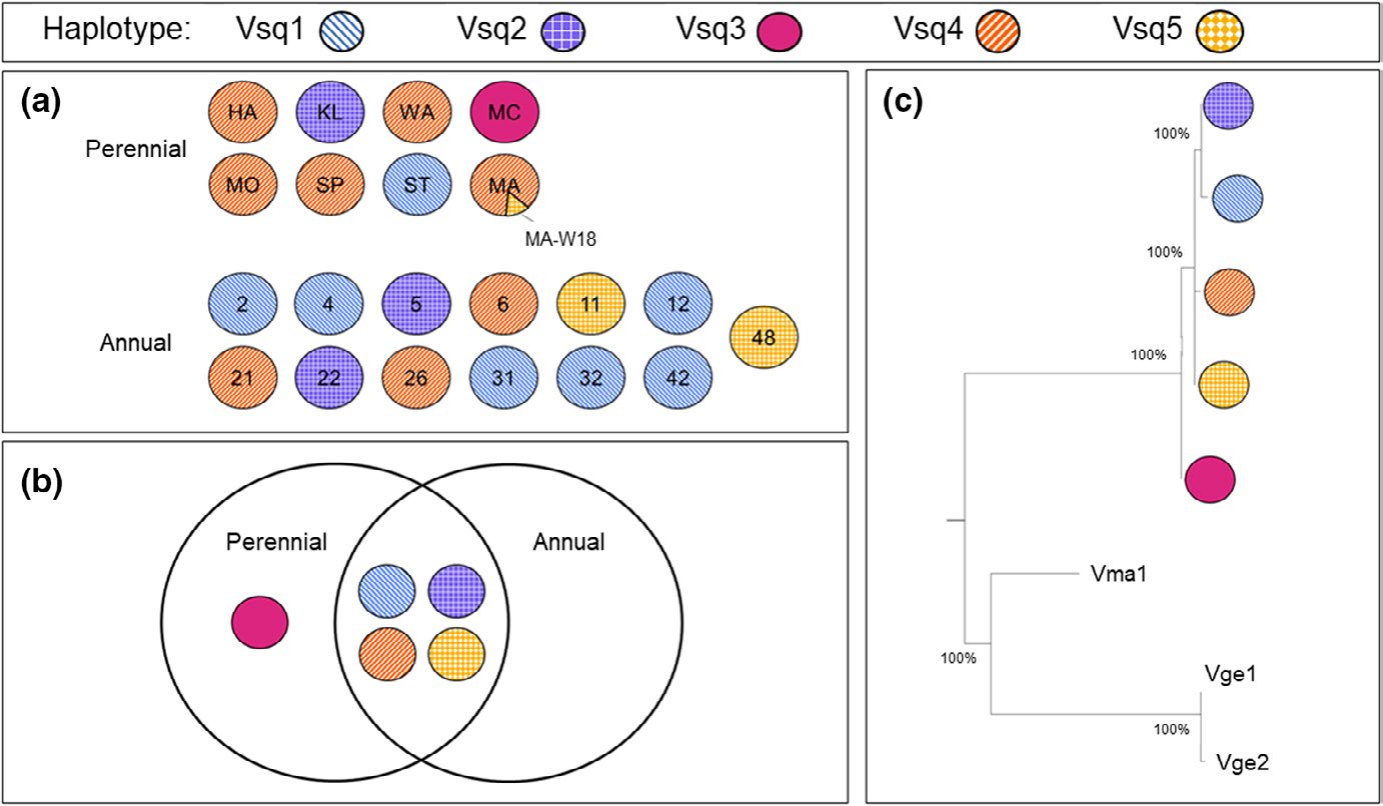
Mitochondrial haplotypes for cytochrome b in *Vespula squamosa*. (a) Haplotype frequencies within each colony. (b) Distribution of haplotypes in annual and perennial social forms. (c) Maximum‐likelihood phylogeny of five mitochondrial haplotypes with *Vespula germanica* (Vge) and *Vespula maculifrons* (Vma) outgroups

We identified a total of five variable bases within our trimmed 338 bp sequence of the cytochrome b gene, indicating the presence of five unique haplotypes in our sequenced *V. squamosa* samples. Four of the haplotypes were found in the annual samples, while all five were present in the perennial samples (Figure 7a,b). Overall, we calculated a total haplotype diversity in our *V. squamosa* samples of 0.6116. Annual colonies (0.7267) had a similar haplotype diversity to perennial colonies (0.5641).

All annual colonies displayed a single mitochondrial haplotype, which is to be expected in a closed system with a single reproductive queen. Notably, however, we also found that all individuals from 7 out of 8 perennial colonies possessed only a single haplotype, suggesting that all individuals belonged to a single matriline. The only multiple‐haplotype perennial colony (MA) contained a single worker that differed in haplotype from the rest of the sampled individuals in that colony. This haplotype was confirmed by re‐sequencing the individual in question.

A maximum‐likelihood phylogeny was generated to understand the relationships among the five different haplotypes (Figure 7c). We found that the five haplotypes identified in *V. squamosa* sorted into a monophyletic group when compared to the sequences from the outgroups *V. maculifrons* and *V. germanica*. We also investigated whether the social forms showed significant differences in mtDNA haplotype frequency using an abbreviated dataset consisting of only a single individual per colony. We found no significant differences in haplotype frequency between social forms using a test of genic differentiation (*p* = .2077).

## 4 DISCUSSION

The goal of this study was to gain a greater understanding of variation in the social structure of a highly social insect. We specifically investigated the social systems of large, perennial *V. squamosa* colonies. Our goal was to understand how the social structure of perennial colonies differs from that of typical annual colonies and provide insight into future changes in social behavior.

### 4.1 Reproduction and recruitment within perennial colonies

We found that *V. squamosa* perennial colonies were always headed by multiple queens. The genotypes of sampled workers, gynes, and males were consistent with reproduction by multiple reproductive females. This was supported by maximum‐likelihood estimations of queen number that indicated that colonies could be headed by 20 or more queens. However, we note that the exact assessments of queen number are likely to be complicated by queen polyandry, suggesting that actual estimates of queen number should be viewed cautiously. Regardless, our data clearly demonstrate that perennial colonies are headed by many reproductive queens. This differs substantially from annual *V. squamosa* colonies, which are always headed by a single queen that produces all workers, gynes, and males as long as she is present (Hoffman et al., 2008). This variation in colony queen number represents an important change in life history and social biology (Crozier & Pamilo, 1996; Keller, 1993).

Multiple‐queen (polygyne) *V. squamosa* colonies could arise through one of two mechanisms. First, new queens could be recruited from within their natal nests. Alternatively, foreign queens from unrelated *V. squamosa* colonies could enter into already established colonies and begin producing new progeny. One way to distinguish these mechanisms is to study patterns of maternally inherited genetic markers, such as the mtDNA (Goodisman & Ross, 1998). If new queens are recruited from their natal nest, then nestmates should always possess only a single mtDNA haplotype (i.e., that of the original mother queen). However, if foreign individuals enter nests to reproduce, then individuals from a single colony may contain multiple mtDNA haplotypes representing the matrilines of each new queen.

The overall result from our analysis of mtDNA was that workers, gynes, and males from perennial nests almost always possessed a singular mtDNA haplotype. There was a single worker from one perennial colony (MA) that possessed a mtDNA haplotype inconsistent with the rest of the colony. The importance of this individual is hard to interpret, as it could represent a rare event such as worker drift or even cross contamination. Therefore, overall, the data suggest that members of *V. squamosa* perennial colonies, including the multiple female reproductives, generally originate from their own parental nest.

The finding that perennial *V. squamosa* colonies recruit nestmate queens fits with general expectations from kin selection theory (Crozier & Pamilo, 1996). That is, cooperation among individuals is expected to occur between relatives (Bergmuller et al., 2007; Sachs et al., 2004). *Vespula squamosa* workers can produce males if the colony loses its queen. But they do not mate and cannot produce female offspring. Therefore, they generally do not gain direct fitness benefits by producing their own offspring. Instead, they receive indirect benefits by helping to rear relatives produced by the queen. Cooperation and reproductive altruism can only evolve if nestmates are related (Kay et al., 2020). Thus, the finding that *V. squamosa* are mostly closed societies fits with these expectations as the introduction of foreign queens into the colony would cause a decrease in relatedness, and thus indirect fitness, overall.

Although new *V. squamosa* queens were recruited from within their natal nests, the male mates of new queens were apparently not. Genetic analyses indicated that perennial colonies contained a substantial increase in the number of nuclear alleles compared to annual colonies. This increase in rare alleles was associated with a decrease in nestmate relatedness. Thus, there was apparent gene flow into perennial colonies. In particular, new queens presumably mated with non‐nestmate males. However, these mated queens apparently returned to their natal nest to reproduce. *Vespula* are capable of inbreeding in the laboratory (Kovacs et al., 2008). However, *Vespula* rarely inbreed in natural circumstances and have evolved several mechanisms to avoid inbreeding (Goodisman et al., 2002; Martinez et al., 2018, 2021; Masciocchi et al., 2018, 2020). Thus, perennial *V. squamosa* colonies increase in genetic diversity over time through queen outbreeding, which leads to lower nestmate relatedness overall.

Interestingly, previous studies of perennial colonies in other *Vespula* species have uncovered evidence for at least occasional queen recruitment from outside the nest (Gambino, 1991; Goodisman, Matthews, Spradbery, et al., 2001; Hanna et al., 2014; Loope et al., 2018; Scarparo et al., 2021). Thus, different *Vespula* species show variation in whether they accept non‐nestmate queens into the colony (Loope et al., 2018). Importantly, the loss of colony boundaries associated with non‐nestmate recruitment and recruitment of multiple queens has been identified in other social insects, most notably in many invasive ants (Helantera et al., 2009; Suarez & Goodisman, 2021). Such breakdowns are associated with changes in environmental conditions and, perhaps, with genetic changes to populations.

### 4.2 Reproductive competition within perennial colonies

The presence of multiple reproductive queens within *V. squamosa* perennial colonies raises the possibility that queens may engage in various types of reproductive competition with each other (Foster & Ratnieks, 2001; Ratnieks et al., 2006; Wenseleers et al., 2004). For example, prior studies have found that nest cells sometimes hold multiple eggs, indicating a breakdown of colony reproductive integrity (Kovacs & Goodisman, 2007; Spradbery, 1973). We investigated whether the genotype distribution of gynes differed from that of workers in perennial colonies. Such differences could arise if different queens, or queens’ male mates, contributed differentially to gyne and worker production (Boomsma et al., 2014; Heinze, 2010; Ratnieks et al., 2006) or if there were some other genetic effects on caste formation (Anderson et al., 2008; Lo et al., 2009; Schwander et al., 2010).

Interestingly, we found evidence of genetic differences between gynes and workers in three of the six colonies analyzed. This indicates that different queens or males likely produced the two castes in these colonies. Our sample size was relatively small for these analyses and so some caution is warranted in interpreting the results. However, secondary significance tests supported the results of the analyses that there were differences in the genotypes between castes in perennial nests. Therefore, reproductives in polygyne *V. squamosa* nests apparently contribute differentially to the different castes, but more rigorous analysis would be needed to elucidate the details of the system.

Prior studies have found that genotype can affect caste phenotype in *Vespula* (Kovacs & Goodisman, 2012; Kovacs et al., 2010; Perrard et al., 2012). However, a previous investigation in *Vespula* found no evidence for different patriline contributions to gynes and workers (Goodisman, Matthews, & Crozier, 2007). Therefore, it is more likely that queens within perennial colonies contribute differentially to gyne and worker production. One might expect that queens within perennial colonies would compete to produce gynes rather than workers, as gyne production would presumably lead to larger increases in direct fitness, since workers can only produce males under restricted circumstances. Such reproductive competition should be an important factor affecting behavioral evolution (Ratnieks et al., 2006; Tarpy et al., 2004). It thus appears that reproductive competition may take place in *V. squamosa* perennial colonies among different queens (Stewart et al., 2017).

We identified a single perennial colony that generated a putative satellite colony nearby to the main nest. This appeared to be an instance of incipient polydomy, which is the occupation of multiple nests by a single colony. Polydomy is more often associated with terrestrial social insects such as ants or termites (Debout et al., 2007; Ellis et al., 2017; Robinson, 2014). However, polydomy could, in principle, occur in wasps as well. We found that there were large differences in the genotypes of males and workers sampled from the bud and parent colony, but these differences were not statistically significant and were limited in the samples that could be collected for analysis. Nevertheless, it is intriguing to consider if these polygyne perennial colonies can create buds that ultimately become genetically differentiated from their parent colony.

### 4.3 Genetic differences between perennial and annual colonies

We sought to investigate whether the formation of perennial *V. squamosa* colonies represents true evolution (i.e., genetic change) or phenotypic plasticity. We attempted to explore this question by testing for genetic differences between the nuclear and mtDNA genotypes of annual and perennial colonies. We found no evidence of genetic differences between the social forms at either set of markers. In addition, there was no evidence of genetic isolation by distance within the social forms. Thus, overall, we find no evidence of genetic differentiation among the social forms. We note that this analysis, which includes the use of only a few genetic markers, is insufficient to provide a strong test of genetic differentiation between social forms. Nevertheless, our analysis can be viewed as providing preliminary insight into the question of whether annual and perennial *V. squamosa* colonies belong to the same gene pool, and our data are consistent with the idea that the perennial colonies represent an instance of phenotypic plasticity rather than evolution.

Previous investigations of other social species have sometimes uncovered evidence for a genetic basis to complex social behavior (Gutierrez‐Valencia et al., 2021; Schwander et al., 2014). For example, variation in social form in two different ant genera has a genetic basis (Brelsford et al., 2020; Wang et al., 2013). Thus, phenotypic plasticity in social behavior may be associated with traits becoming genetically fixed. Or mutations may arise that lead to variation in complex social behavior (Rubenstein et al., 2019). Our research on *V. squamosa* social forms has not uncovered evidence of genetic differences at this time. But more in‐depth genomic analysis is needed to determine whether such genetic differences exist or whether they are likely to arise in the future.

### 4.4 Future of *Vespula* perennial colonies

Perennial colonies are found in several *Vespula* taxa (Gambino, 1991; Goodisman, Matthews, Spradbery, et al., 2001; Hanna et al., 2014; Jeanne, 1980; Lester & Beggs, 2019; Loope et al., 2018; Loope & Rankin, 2021; Plunkett et al., 1989; Reed & Landolt, 2005; Ross & Visscher, 1983; Spradbery, 1991; Thomas, 1960; Visscher & Vetter, 2003; Wilson et al., 2009). These large, persistent colonies in other *Vespula* are often associated with invaded habitats (Eyer & Vargo, 2021). Therefore, it is notable that *V. squamosa* forms perennial colonies in its native habitat, but only in warmer regions.

The perennial colonies of *V. squamosa* are thought to have a substantial ecological effect on the local environment. Their large size means that they take many more prey than a typical annual nest. Additionally, they become much more of a human nuisance because they contain orders of magnitude more workers than a typical nest (Beggs et al., 2011; Lester & Beggs, 2019; Wilson & Holway, 2010; Wilson et al., 2009). *Vespula* perennial colonies are expected to increase their range in response to climate change, which will further increase negative interactions with humans (Demain, 2020; Komonen et al., 2020; Lester et al., 2017). Moreover, it has been found that relatively few *Vespula* wasps are needed to initiate a new introduced population (Brenton‐Rule et al., 2018; Chau et al., 2015; Dobelmann et al., 2019; Eloff et al., 2020; Hanna et al., 2014; Schmack et al., 2019). Thus, *Vespula* wasps have had great success in both introduced and native populations around the world (Beggs et al., 2011; Lester & Beggs, 2019; Lowe et al., 2000; Manfredini et al., 2019). It is possible that continued global warming combined with increased movement of propagules will lead to *Vespula* perennial colonies worldwide.

## CONFLICT OF INTEREST

The authors declare that they have no competing interests.

## AUTHOR CONTRIBUTIONS

**Carl J. Dyson:** Conceptualization (supporting); Formal analysis (equal); Investigation (equal); Methodology (equal); Writing – original draft (equal). **Henry G. Crossley:** Data curation (equal); Methodology (equal). **Charles H. Ray:** Investigation (supporting); Methodology (supporting); Resources (lead). **Michael A. D. Goodisman:** Conceptualization (lead); Formal analysis (supporting); Funding acquisition (lead); Investigation (equal); Project administration (lead); Resources (lead); Supervision (lead); Validation (lead); Writing – original draft (equal).

## Data Availability

New mitochondrial DNA sequences are available through GenBank accessions MZ820430–MZ820649. The perennial *Vespula squamosa* genotypic data have been deposited in Dryad and are available at https://doi.org/10.5061/dryad.rjdfn2zcg.
